# ACCV: automatic classification algorithm of cataract video based on deep learning

**DOI:** 10.1186/s12938-021-00906-3

**Published:** 2021-08-05

**Authors:** Shenming Hu, Xinze Luan, Hong Wu, Xiaoting Wang, Chunhong Yan, Jingying Wang, Guantong Liu, Wei He

**Affiliations:** 1 College of Medicine and Biological Information Engineering, Northeastern University, Shenyang, 110016 China; 2 He Eye Specialists Hospital, Shenyang, 110000 China; 3 He University, Shenyang, 110000 China; 4 Shenyang Eyerobo Co., Ltd., Shenyang, 110000 China

**Keywords:** Automatic cataract grading, Deep learning, YOLOv3

## Abstract

**Purpose:**

A real-time automatic cataract-grading algorithm based on cataract video is proposed.

**Materials and methods:**

In this retrospective study, we set the video of the eye lens section as the research target. A method is proposed to use YOLOv3 to assist in positioning, to automatically identify the position of the lens and classify the cataract after color space conversion. The data set is a cataract video file of 38 people's 76 eyes collected by a slit lamp. Data were collected using five random manner, the method aims to reduce the influence on the collection algorithm accuracy. The video length is within 10 s, and the classified picture data are extracted from the video file. A total of 1520 images are extracted from the image data set, and the data set is divided into training set, validation set and test set according to the ratio of 7:2:1.

**Results:**

We verified it on the 76-segment clinical data test set and achieved the accuracy of 0.9400, with the AUC of 0.9880, and the F1 of 0.9388. In addition, because of the color space recognition method, the detection per frame can be completed within 29 microseconds and thus the detection efficiency has been improved significantly.

**Conclusion:**

With the efficiency and effectiveness of this algorithm, the lens scan video is used as the research object, which improves the accuracy of the screening. It is closer to the actual cataract diagnosis and treatment process, and can effectively improve the cataract inspection ability of non-ophthalmologists. For cataract screening in poor areas, the accessibility of ophthalmology medical care is also increased.

## Introduction

Cataract as the main blinding eye disease and has a serious impact on people's health and life [1]. The global blindness due to cataract accounts for more than 50%. As a country with the most population, China has about a quarter of the world's visually impaired and blind population [2]. The American Academy of Ophthalmology (AAO) defines cataract as the opacity of the lens [3]. Untreated cataract is still the main cause of blindness in the world, and there are nearly 18 million people who lose sight in both eyes [4]. Through cataract screening, more people accept cataract examination, which has proved to be an effective way to significantly improve blindness. Five years after the implementation of free cataract screening and low-cost cataract surgery in rural areas in southern China, the opportunities of women and illiterate patients receiving surgery have increased [5]. Carrying out intelligent cataract and eye disease examination is the trend of development and the necessary way. Especially with the rapid development of artificial intelligence technology in recent years, the automatic lens image recognition technology has been improved.

However, the current image recognition technology is mainly the recognition of a single lens image, and the early stage mainly relies on manual image grading and labeling, and then model training [6–10]. These methods realize the automatic grading of cataract to some extent and save manpower, but this method still has shortcomings. These methods are mainly used for analysis of single lens images. According to the Tyndall effect, he observes the degree of turbidity in the area covered by the light band and judges whether the subject has cataract or not. However, in reality, the doctor does not perform cataract examination based on a single lens image during the examination, but makes a comprehensive diagnosis after scanning the whole lens and inquiries about the patient’s age, corrected vision, and past medical history.

In all current researches using image algorithms and artificial intelligence automatic grading, the research goals are still limited to a single image, which is far from the real diagnosis and treatment. It is also difficult for doctors to make an accurate diagnosis with only a single lens image. Based on the convenience of community screening, the doctor's actual diagnosis process and the increased input information, this paper adopts a more convenient mobile phone slit lamp that facilitates the screening process, and collects the video of the entire lens as the analytical object [11]. Different from traditional researches based on the single image, this algorithm proposes a real-time lens cataract grading method based on target detection technology. It uses the entire lens as the research target, increasing the amount of input information of the classification algorithm, and optimizing the target detection algorithm, making the screening process more efficient and more in line with the doctor's diagnosis process. Since the LOCS classification method was proposed, computer automatic classification methods have been continuously proposed in recent years. Most methods use the single lens image taken by a desktop slit lamp as the research object.

Researchers in literature [6, 12–15] can effectively extract the features of cataract images and use classification algorithms to achieve automatic grading. The basic principle is to extract the global or local features of the image, and finally use the support vector machine (SVM) to complete the cataract classification task or the support vector regression algorithm (SVR) to complete the grade prediction, and the accuracy rate can reach nearly 90%. Xu et al. [16] used the group sparsity regression to perform feature selection and parameter selection after extracting features from the image, and finally completed the classification. The linear discriminant analysis (LDA) is trained to detect cataracts by using the enhanced texture features of the extracted cataract images and the statistical data of the enhanced texture features [17]. Testing on 4545 medical images can achieve an accuracy of 84.8%. The above-mentioned automatic grading methods have all completed experimental researches using traditional methods. In recent years, with the rapid development of deep learning, research on cataract grading using related algorithms in deep learning has also been rapidly developed. Literature [7] uses convolutional neural network to complete the feature extraction of congenital cataract data set, and combines SVM algorithm or SoftMax classifier to realize automatic classification. At the same time, this method is compared with traditional representative methods. The effect of automatic classification is good. Meanwhile, literature [18] uses the deep convolutional neural network in the field of deep learning to complete cataract classification and detection. This method sets the research goal as fundus images, and the current classification also achieves an accuracy of 86.7%. Literature [19] also extracts the feature from the fundus image. The difference lies in the use of improved Haar wavelet automatic recognition. Some studies have used ultrasound as the detection source, which can measure the cataract hardness characterization while automatically grading [20]. Xu et al. [9] published a paper at the MICCAI conference to better study the classification of nuclear cataracts. By using faster R-CNN to locate the nuclear area and taking the nuclear area as input, the classification based on ResNet-101 model has been trained. In addition to the use of image algorithms, literature [21] uses the next-generation sequencing (NGS) method of DNA to diagnose congenital cataracts.

Although methods for automatic cataract grading are constantly proposed, these methods are still limited to single image. Compared with the doctor's diagnosis and treatment process, the input information is greatly reduced. Through a visit to the doctor, it was found that the ophthalmologist believed that the single-image classification method would lead to misdiagnosis. In this regard, this paper proposes a method that uses the entire eye lens video collected by the mobile phone slit lamp as the research object, and uses the YOLOv3 [22] algorithm to assist in positioning to complete the identification and classification of cataracts. The detailed method is described in the second part of the article.

The structure of the article is as follows. The “Introduction” section introduces the automatic cataract classification algorithms that have been developed so far. The “Method” section introduces the detailed process of this algorithm. The “Results” section provides the research results of this algorithm. The “Conclusion” section summarizes and discusses the application of this algorithm.

## Method

In this retrospective study, we propose a cataract screening method based on video: first, using iSpector Mini to collect crystal data from videos; second, using YOLOv3 to locate the pupil position of the cataract; finally, using the Densenet model to classify the obtained lens images.

### iSpector Mini

**Fig. 1:**
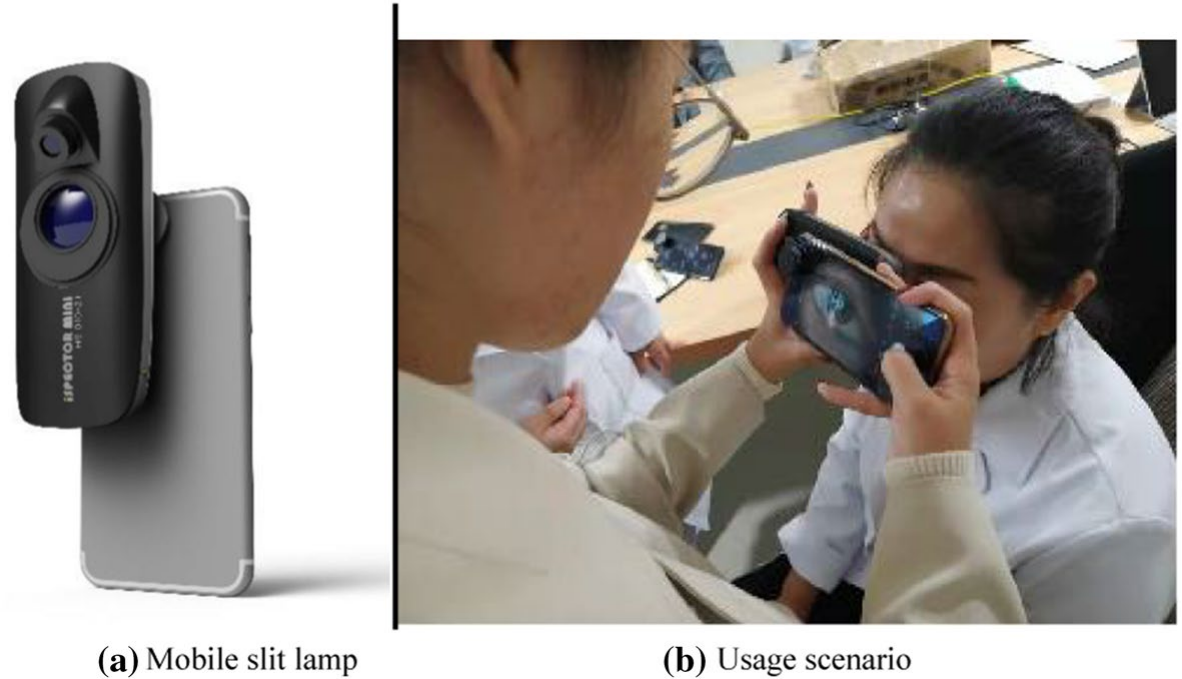
The image and usage scenario of the slit lamp

The equipment used in the video collection is the mobile slit lamp, iSpector Mini, produced by eyeROBO Co., Ltd. The equipment features portability and usability. It is suitable for community screening. Below are its image and usage scenario (Fig. 1).

**Fig. 2:**
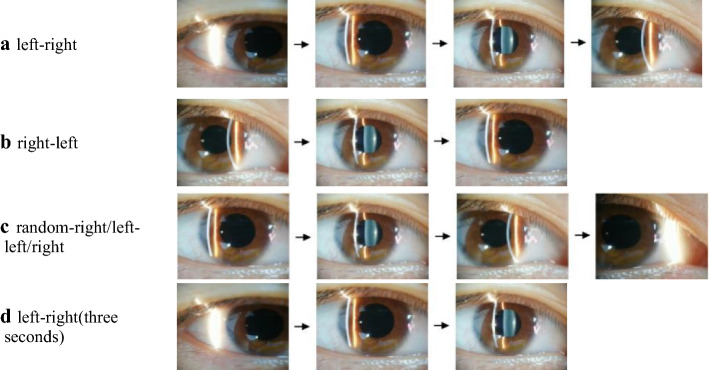
**a–d** Describe the four random collection methods of eye lens, respectively. To reduce the impact of video context correlation on the ACCV method caused by different shooting methods

It is easy-to-use iSpector Mini in community screening in that it can be connected to any phone by the clip. An APP called RUIMU needs to be downloaded in the phone. The app adopts a home-made system and utilizes RUIMU's camera to take the video of crystal when the light projected by the iSpector Mini enters the pupil.

The examinee sits in a chair during the examination, and the examiner instructs the examinee to look straight forward. The examiner adjusts the iSpector Mini to project the light into the pupil and takes the video. At the moment, the video file is automatically recorded in the phone and uploaded to the background server via RUIMU-APP, thus achieving telemedicine. After interpreting the video file into images in this study, relevant algorithms are used to train, verify and test the images.

### Patient dataset

The dataset we use comes from a cooperation hospital using a mobile phone slit lamp to collect eye lens videos. All patients signed a written informed consent form approved by the partner organization. The study was approved by the ethics committee of the partner institution. The data obtained by the partner organization have been de-identified (data have been de-identified). Cataract patients are required to be over 45 years old, and there is no age requirement for normal patients. In this study, the average age of cataract patients was 58 years, and the proportion of men was 60.5% (23 of the 38 people). The collection requires 4 random methods to reduce the impact of video capture on ACCV. The video files by mobile phone slit lamp were collected from 38 people and 76 eyes. The 76 crystal videos collected were then used, including 36 videos of normal eye and 40 videos of cataract eye. A total of 1520 images were interpreted, including 720 normal eye crystal images and 800 cataract eye crystal images. The data set was divided according to a 7:2:1 scale. So 1064 images were training data sets (including 504 normal eye crystal images, 560 cataract eye crystal images) and 304 images were validation data sets (contains 144 images of normal eye crystals, 160 images of cataract eye crystals), 152 images were test data sets (including 72 images of normal eye crystals, 80 images of cataract eye crystals).Organize the video files and manually grade the eyes of the patients in the video according to the advice from three doctors of the partner hospital to confirm whether they have cataracts. One doctor is the chief physician with more than 10 years’ experience in cataract diagnosis. The other two doctors have more than 5 years’ experience of cataract-related work. Ophthalmologists voted to determine the final classification of some controversial images. The images will eventually be classified into two categories: cataract and normal. The labeling software uses LabelImg, and the software version is 1.8.1. The equipment used to collect data is the iSpector-mini mobile phone slit lamp developed by Shenyang EyeROBO Intelligent Technology Co., Ltd., and the mobile phone is iPhone 7. The reasons we use the slit lamp are as follows. This slit lamp is easy to operate and obtain the video eye data of the subject, as well as the patient well-accepted it. And it is more suitable for screening. The capture videos are within 10 s.

The four random collection methods are as follows:

1. As shown in Fig. 2a, the fissure image moves from the left sclera of the subject through the left iris–pupil–right iris, and stops at the right sclera.
2. As shown in Fig. 2b, the fissure image moves from the right sclera of the subject through the right iris–pupil–left iris and stops at the left sclera.
3. As shown in Fig. 2c, the fissure image starts at a random position in the iris, passes through the left/right iris–pupil–right/left iris, and stops at the right/left sclera.
4. As shown in Fig. 2d, the fissure image enters from the left sclera, passes through the left iris to the pupil and lasts for three seconds.

Repeat (1) and (2) three times. The data collection method of 38 people is randomly selected from the above 4 methods in order to reduce the impact of video context correlation on the ACCV method caused by different shooting methods.

**Fig. 3:**
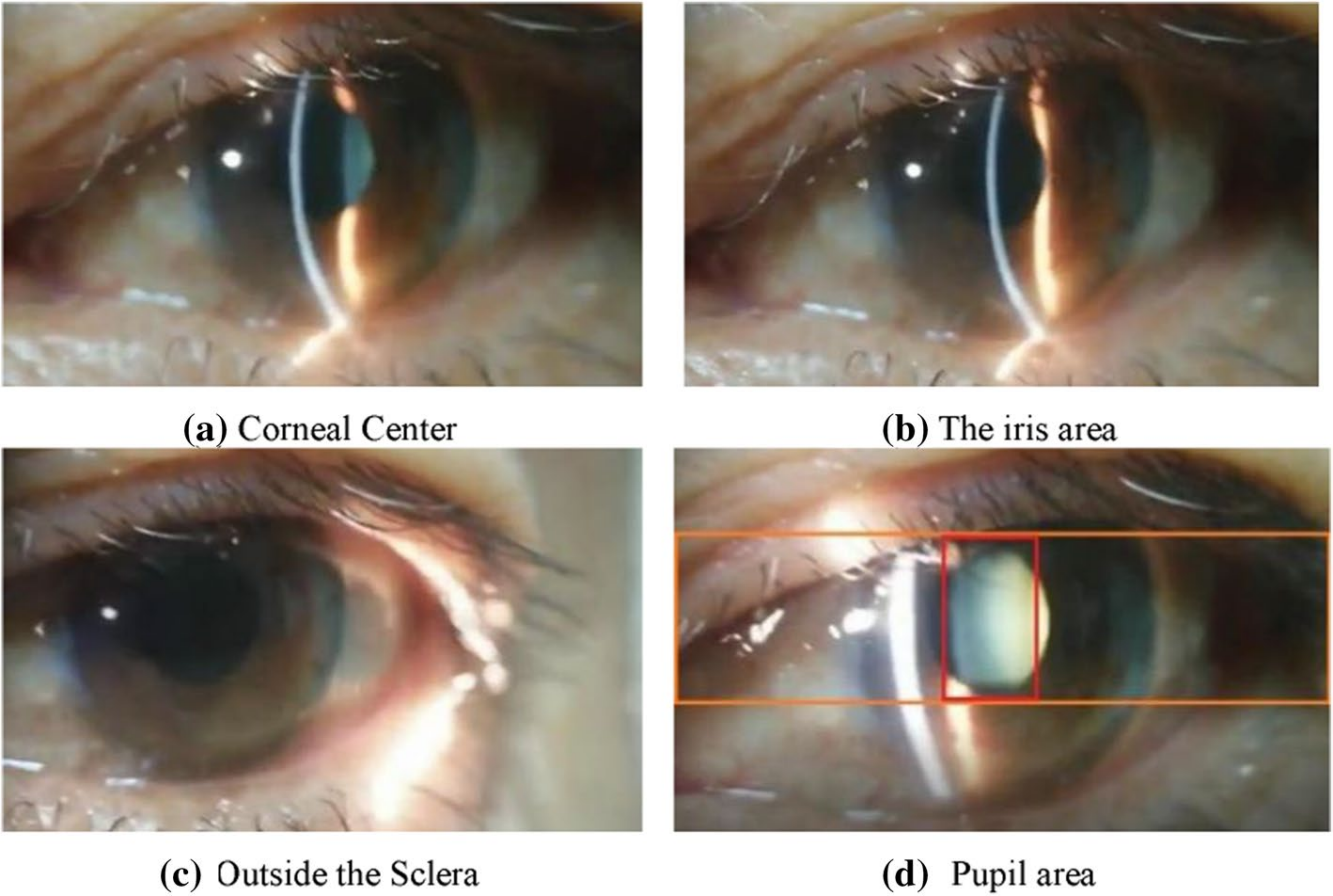
Example image of a light knife cutting into the lens area. **a–c** are diagrams when the slit image is outside the pupil; **d** within the red frame, the light knife can be considered to have entered the pupil area; while within the orange frame, the light knife is considered to be outside the pupil

As shown in Fig. 3, it is an example of the light knife cutting into the eye lens. Within the red frame, the light knife can be considered to enter the pupil area; and within the orange frame, the light knife is considered to be outside the pupil. If two consecutive frames are recognized by YOLOv3, it is considered that the slit light knife has entered the pupil area. And the two consecutive frames aim to remove the impact of random interference. At this time, the next 5 frames are continuously extracted and sent to the YCrCb space for the next auxiliary judgment.

**Fig. 4:**
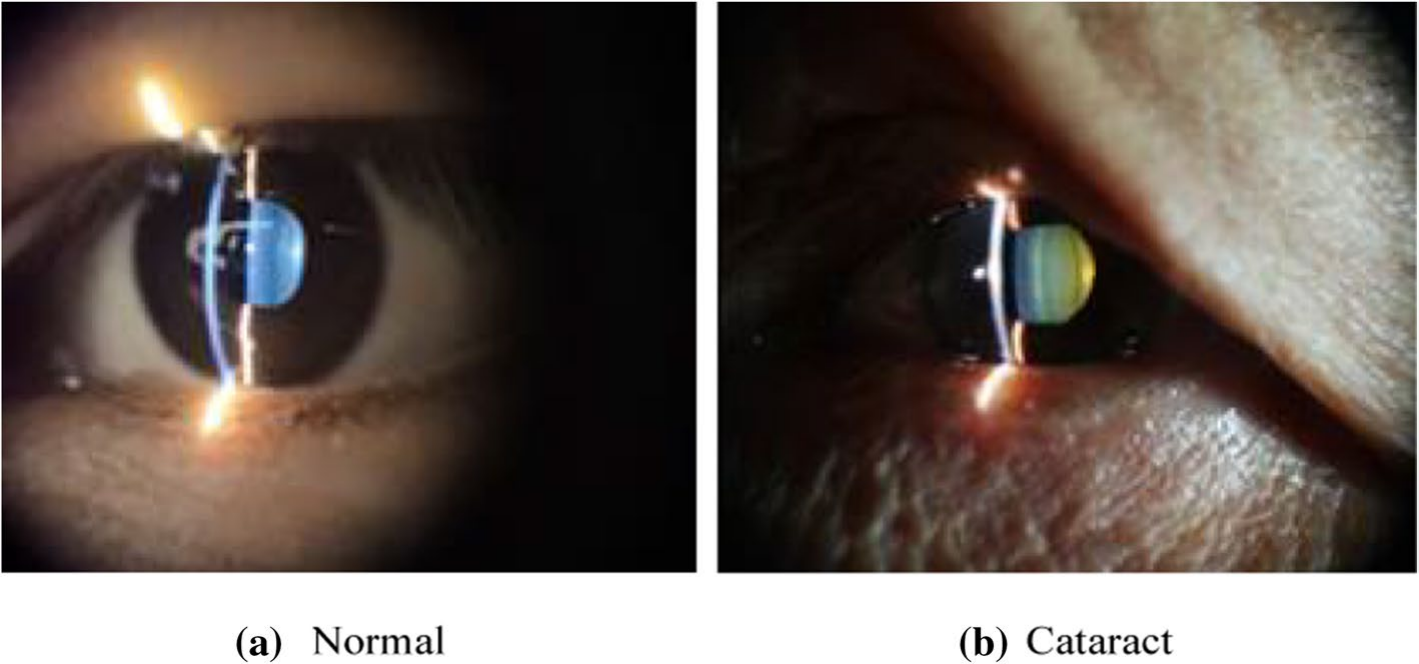
Sample of lens image classification marked by hospital doctors

From Fig. 3, a–c are the images when the light band does not enter the pupil, and (d) is the image when the light band enters the pupil. Observing changes in the pupil area, we can see that after the slit light enters the pupil, the image brightness and saturation in the pupil will change significantly. If you use YOLOv3 for pre-position to obtain the pupil area information, you can use the Cb signal in the YCrCb to assist YOLOv3 in positioning. Cb reflects the difference between the blue part of the RGB input signal and the RGB signal brightness, which significantly increases the detection speed, and reduces the delay in the recognition process and the dependence on the hardware operation speed, as well as improves the overall real-time performance and availability (Fig. 4).

### YOLO

YOLO (You Look Only Once) is a detection method that directly uses the idea of regression to perform frame selection, that is, get the classification and probability directly from the image pixels. The core idea is to use the entire picture as the input of the network. Return the position of the bounding box directly in the output layer to achieve end-to-end target detection. The main steps of feature extraction are as follows:

1. Divide the whole picture into *S* × *S* grid cell. If the center of an object falls in a grid cell during the recognition, then the corresponding grid cell is used to predict the object.
2. If the center of the measured object falls in a grid cell, the corresponding grid cell starts to work, extract the feature of the target. Each grid cell will predict the bounds of b and the probability of each object and its offsets.
3. b bounding boxes identify b rectangular regions. Each area indicates a message, and the probability of the detected object corresponding to the object information.
4. Center at the grid cell. 5-bit parameters of each frame need to be predicted. YOLO finds 2 bounding boxes and gives two prediction results. The 5 parameters are *x*, *Y*, *h*, *W* and confidence. The coordinates plus the confidence are 5 bits in total, and each grid cell predicts 2 bounding boxes, which are 10 bits. Also output the probability C that each grid cell predicts an object. It can be known that each bounding box output confidence and probability are a tensor of S*S*(5*b + *c*).

### Overall structure

**Fig. 5:**
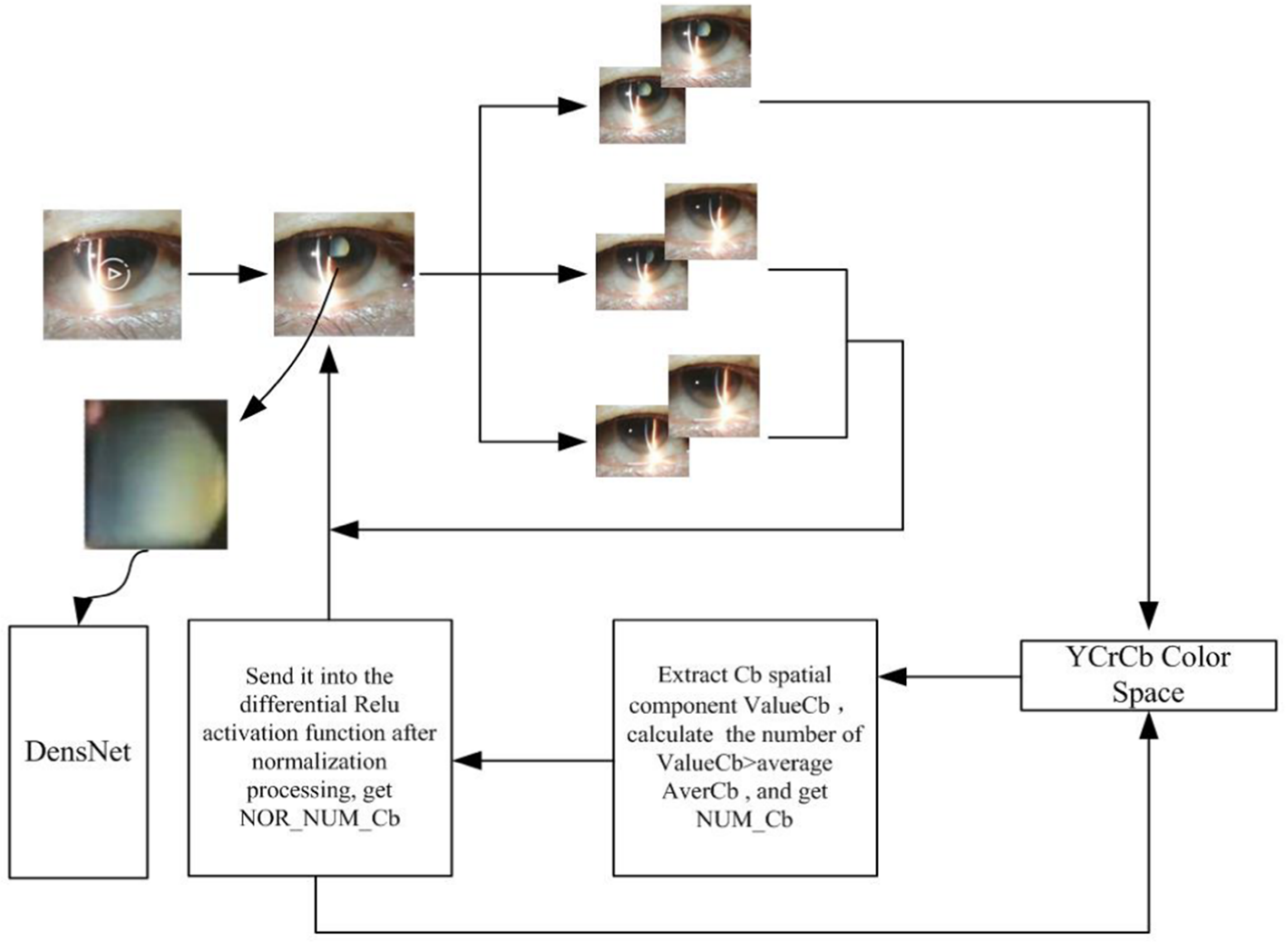
The overall flowchart of the ACCV method

ACCV reads the video of the anterior segment under the slit lamp of the mobile phone, and sends each frame of the video to YOLOv3 for real-time recognition. The recognition process has completed two tasks. The first is to cut out the gaps in the captured video files. The second is to complete the automatic classification. If the pupil area is not recognized, the detection of YOLOv3 is repeated continuously, and ACCV does not do any other processing during the detection. The specific process of the proposed method is shown in Fig. 5 (Table 1).

### YOLOv3 pupil detection

Send the obtained video file to YOLOv3 for judgment. In the continuous recognition process, if two consecutive frames are recognized by YOLOv3 as in the pupil area, then the second frame is sent to the Cb space to obtain Value Cb, the Cb of each pixel. Count the number of pixels whose ValueCb is greater than the average value to obtain Num_Cb. Use formula:

1 $${y}_{i} = \frac{{x}_{i}}{\sum_{i=1}^{n}{x}_{i}}.$$

Normalize Num_Cb and obtain the pixel number Nor_Num_Cb. The formula of Relu activation function is:

2 $$\text{ReLU} = \left\{ {\begin{array}{*{20}l} {x\quad if\;x > 0} \hfill \\ {0\quad if\;x \le 0} \hfill \\ \end{array} .} \right.$$

Here, the differential Relu activation function is used to increase the degree of non-linearity of the algorithm and make the expression of the algorithm more specific. After being processed by the differential Relu activation function, a binarized graph is obtained, whose gray level is greater than the average in the Cb space. As shown in Fig. 3, we know that when the Cb quantity space is in the zero area, the light knife is in the pupil, which is basically the same as the pupil determined by YOLOv3. If the Cb quantity space is located in area 1, it means that the light knife has not entered the pupil, and it is in the area outside the pupil. The reason is that after the slit image enters the pupil, the difference between the blue part of the RGB input signal represented by the Cb space in the pupil and the brightness value of the RGB signal changes sharply. Therefore, when we continuously take 5 frames of images, we must use the Cb space as a reference. If the Cb value of these 5 frames of images is zero, it means that the light knife is still in the pupil, and the classifier can be called directly for classification instead of calling the YOLOv3 locator, thereby improving the operating efficiency of the entire system. If the Cb quantity space is located in area 1, stop the continuous call of 5 frames of images, return to the YOLOv3 algorithm, and call YOLOv3 again to determine whether it has been moved out of the pupil.

**Fig. 6:**
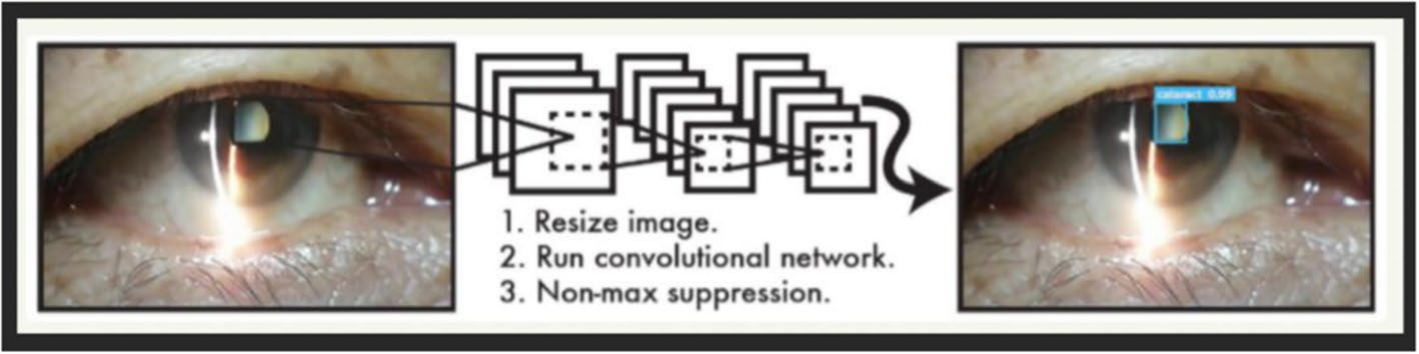
Basic principles of YOLOv3 diagram

As shown in Fig. 6, the CNN network divides the input picture into *S* × *S* grids, which are called bounding boxes. Then each cell is responsible for detecting those targets whose center point falls within the grid. All bounding boxes are sent to the classifier for classification. The image characteristic of cataract video detection is that when the patient is fixed, the position and size of the lens section in the image are basically unchanged. So the proposed method uses YOLOv3 to assist in determining the position of the eye lens section. Then stop the call of YOLOv3 in the consecutive multiple frames. By the color space determination, the algorithm has the prior knowledge of the identification information obtained by YOLOv3. Under the premise, the connection between the contexts of the video is used to continuously predict the position of the eye lens, thereby improving the judgment efficiency of the system. Figure 8 gives the data and description of the YCrCb domain.

**Fig. 7:**
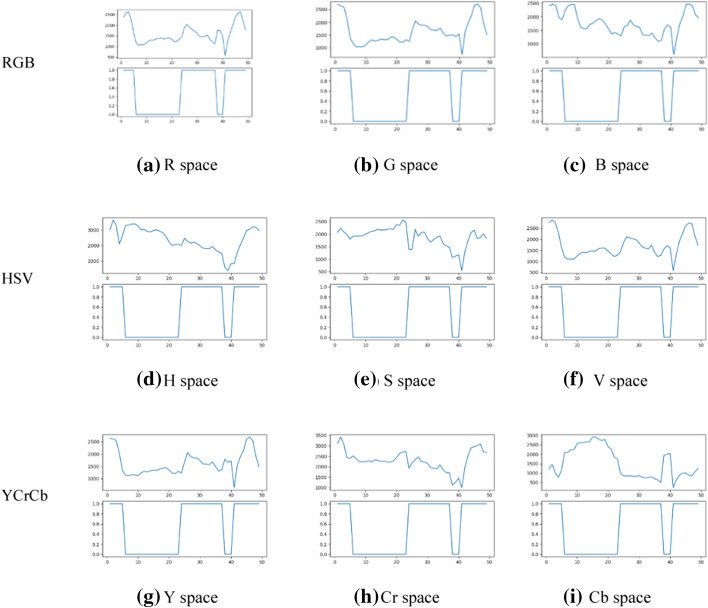
Each color interval of binarized graphs greater than the average gray level. The change in Cb space is closest to whether the slit light is in the pupil

As shown in Fig. 7, after locating the pupil area in the same eye lens video image YOLOv3, the pixels of the color space over than the average gray level is gathered. This solution compares all the commonly used color spaces in RGB space, HSV space and YCrCb space. It is found that the Cb signal that can reflect the difference between the blue part of the RGB input signal and the brightness value of the RGB signal is closer to the change trend of the number of pixels. Replace the positioning result of YOLOv3 with the change of Cb space.

The way of using color space is not absolute. It will be affected by many conditions including ambient light and corneal reflection. Therefore, the method uses the Cb space-assisted screening. Under the condition that the Cb space corresponds to the pupil fan area, after every 5 frames of inference, it is necessary to return to YOLOv3 for re-recognition, that is, the position and size of the pupil area deduced in each Cb space are only valid in the last 5 frames. If it exceeds 5 frames, you must go back to YOLOv3 and continue to judge again to ensure that the position and size of the ROI area can always indicate the position and size of the pupil fan area.

### Eye lens classification

The pupil area recognized by YOLOv3 is intercepted from the original image, and the obtained image data set is classified based on DenseNet with deep learning.

In a traditional convolutional neural network, if you have an *L* layer, then there will be *L* connections. But in DenseNet, there will be L*(*L* + 1)/2 connections. Unlike ResNets, DenseNets do not add features before passing them to a layer, but stitch them together. Therefore, the first layer has one input. These inputs are the feature maps of all convolutional blocks before this layer, and its own feature map is passed to all subsequent layers. The advantage of DenseNet is that it reduces the disappearance of gradients. This connection makes the transfer of features and gradients more effective, and the network easier to train. At the same time, the transfer of features is strengthened, and the output feature map of each layer of features is used more effectively as the input of all subsequent layers. And to some extent, the number of parameters is reduced.

Based on the above advantages, we adopted the Densenet as the final classification network of ACCV. Experiments have proved that a good classification effect is achieved.

During the experiment, we need to constantly adjust the parameters and loss function to get better results. The loss function and various parameters used throughout the experiment are derived from a large number of experiments.

Densnet uses softmax + cross entropy as the loss function, the formula is:

3 $$\mathrm{Cross\;Entropy}=-\sum_{i=0}^{n}p\left({x}_{i}\right)\mathrm{ln}\left(q\left({x}_{i}\right)\right).$$

*p*(*x*) is the true probability distribution, *q*(*x*) is the predicted probability distribution. Cross entropy reduces the difference between two probability distributions to make the predicted probability distribution as close as possible to the real probability distribution.

In practice, it is necessary to use the predicted probability to fit the true probability. At the same time, reduce the impact of over-fitting and artificial labeling errors, thus *p*(*x*) needs to be changed:

4 $$p^{\prime}(x) = (1 - \varepsilon )\delta_{(k,y)} + \varepsilon u(k).$$

*δ*(*k*,*y*) is the Dirac function, and *u*(*k*) is the uniform distribution. In short, reduce the confidence of label *y* and increase the confidence of other categories. Thus, the cross entropy becomes:

5 $$H(p^{\prime},q) = - \sum\limits_{i = 1}^{n} {p^{\prime}(x_{i} )\ln (q(x_{i} ))} = (1 - \in )H(p,q) + \in H(p,u).$$

In the YOLOv3 training, the loss function generally consists of two parts, classification loss and bounding box regression loss. The purpose of classification loss is to make the category classification as correct as possible; the purpose of bounding box regression loss is to make the prediction box match with the ground truth box. Focal loss function is mainly used to solve the imbalance between positive and negative samples. By reducing the loss value in easy classification, the weight of the loss value in difficult classification is indirectly increased. Focal loss is improved based on cross entropy:

6 $${\text{Focal}}\;{\text{loss}} = - \alpha (1 - p_{{\text{t}}} )^{\gamma } \log (p_{{\text{t}}} ).$$

It can be seen that (1 − *p*_t_)*γ* is added before the cross entropy. When the picture is misclassified, *p*_t_ will be small, and (1 − *p*_t_) is close to 1, so the loss is not affected greatly. The addition of the parameter *γ* is to smoothly reduce the weight of the easy example. When *γ* = 0, focal loss degenerates into cross entropy.

At the same time, during training, the networks are trained using stochastic gradient descent (SGD). The dataset we train using batch size 64 for 300 epochs. The learning rate is set to 0.01 initially, and is lowered by 10 times at epoch 30 and 60. Note that a naive implementation of DenseNet may contain memory inefficiencies. We use a weight decay of 10 − 4 and a Nesterov momentum of 0.9 without dampening.

## Results

### Evaluation metrics

In order to evaluate our proposed architecture, the proposed architecture is proposed compared with methods in other papers. As a method of comparison: VGG19, Inception-v3, Resnet50, Mobilenet, Xception. In order to ensure the consistency of the experimental results, the image data set obtained after YOLOv3 eye lens identification is selected as the data set of the classification experiment. At the same time, select the following evaluation indicators as the criteria for judging whether the classification of the same data set under different methods is good or bad. The quantitative indicators we choose are: mAP (average accuracy), Acc (accuracy rate), precision, specificity, sensitivity, F1 value, ROC curve and calculated the AUC index of the area. The calculation formula is as follows:

7 $${\text{Acc}} = \frac{{\left( {{\text{TP}} + {\text{TN}}} \right)}}{{({\text{TP}} + {\text{FN}} + {\text{TN}} + {\text{FP}})}},$$

8 $${\text{Specificity}} = {\text{TNR}} = \frac{{{\text{TN}}}}{{{\text{FP}} + {\text{TN}}}},$$

9 $${\text{Precision}} = \frac{{{\text{TP}}}}{{{\text{TP}} + {\text{FP}}}},$$

10 $${\text{Sensitivity}} = {\text{TPR}} = {\text{Recall}} = \frac{{{\text{TP}}}}{{{\text{TP}} + {\text{FN}}}},$$

11 $$F1 = \frac{{2 * {\text{Precision}} * {\text{Recall}}}}{{{\text{Precision}} + {\text{Recall}}}}.$$

TP, FP, TN and FN stand for the number of true positives, false positives, true negatives and false negatives in the detection results, respectively.

To evaluate the performance of our proposed architecture, select 5 popular deep learning classification networks, which are VGG19, Inception-v3, Resnet50, Mobilenet, Xception, and compare DensNet with these networks.

The evaluation indexes of the algorithm in this paper and the five comparison algorithms are calculated, respectively, and the comparison results are shown in Table 2.

**Table 1:** ACCV algorithm description

Algorithm description
S1. Input a video file collected by the mobile phone slit lamp. Send it to YOLOv3 to identify whether the current frame contains lens section information. If not, continue to identify. If it contains lens section information, then go to the next step
S2. After identifying the information of the lens section, continuously judge whether the next frame also is the lens position to eliminate misjudgment. If two consecutive frames are in the pupil, then go to the next step; if the first frame of YOLOv3 is identified to be in the pupil and the second frame not, or both frames are not in the pupil, then it continues to be sent to YOLOv3 for recognition
S3. After judging that two consecutive frames are in the pupil, and the area shall be sent into the YCrCb space. Take the Cb component to get ValueCb, calculate the number of ValueCb > average AverCb, and get NUM_Cb. And send it into the differential Relu activation function after normalization processing, and unify the different input ranges to get NOR_NUM_Cb
S4. At this time, judge whether NOR_NUM_Cb is zero or not. If it is zero, the demarcated area is still in the pupil, and you can continue to obtain the lens section view. If it is 1, the demarcated area is not in the pupil and then resend it into YOLOv3 to judge whether to enter the pupil area again
S5. The pupil area recognized by YOLOv3 is intercepted from the original image, and the obtained image data set is classified based on DensNet with deep learning

**Table 2:** Comparison of evaluation indexes of VGG19, Inception-v3, Resnet50, Mobilenet, Xception and ACCV

Method	Acc	Sensitivity	Specificity	Pre	F1
ACCV	0.9400	0.9200	0.9600	0.9580	0.9388
Mobilenet	0.8800	0.8200	0.9400	0.9318	0.8723
VGG-19	0.8700	0.7600	0.9800	0.9744	0.8539
Inception-v3	0.8100	0.6600	0.9600	0.9429	0.7765
ResNet-50	0.8600	0.8000	0.9200	0.9091	0.8511
Xception	0.8600	0.8200	0.9000	0.8943	0.8542

**Fig. 8:**
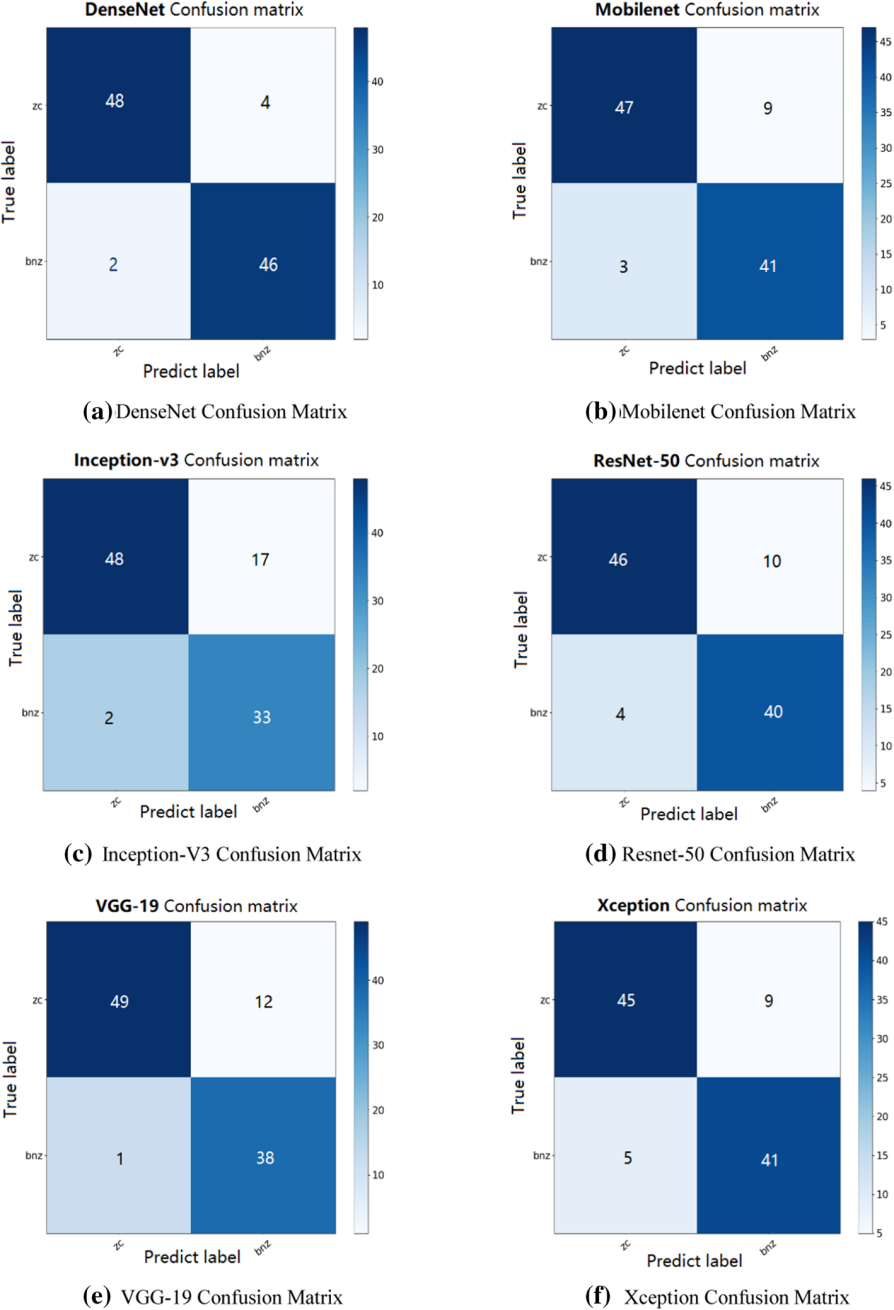
Comparison algorithm and confusion matrix of ACCV

**Fig. 9:**
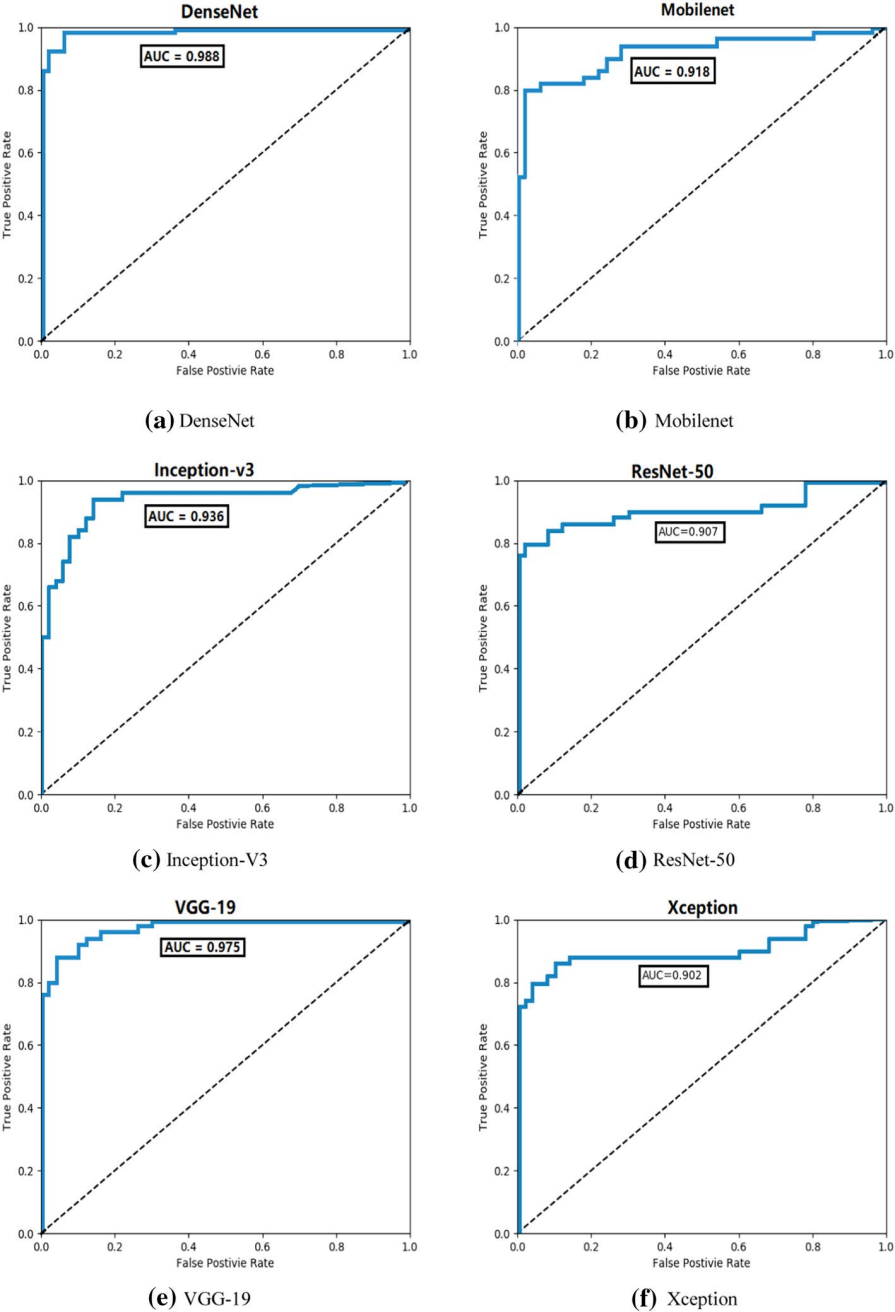
ROC curves and AUC values. **a** Densenet—the classification model in ACCV;  **b** Mobilenet-the classification model; **c** Inception-v3-the classification model; **d** ResNet-50-the classification modelthe classification model; **e** vgg-19-the classification modelthe classification model; **f** Xception-the classification modelthe classification model

In order to evaluate the model's prediction of the classification results, we also measured the ROC curve and calculated the AUC index of the area under the curve of the test dataset. A larger value of AUC has better predictability. At the same time, the predictions are also displayed in the form of a confusion matrix. The ROC curve, AUC value and confusion matrix of the method and comparison algorithm are shown in Figs. 8 and 9.

Figure 8 shows the confusion matrix diagrams of the six methods, including VGG19, Inception-v3, Resnet50, Mobilenet, Xception and ACCV, which are the classification results of each comparison algorithm and the ACCV algorithm. It can be seen from the figure that in the classification, the ACCV algorithm has very few wrong classifications, so the classification can be done accurately and effectively. Figure 9 shows the ROC curve results of the 6 methods including VGG19, Inception-v3, Resnet50, Mobilenet, Xception and ACCV. We can see that the AUC values of these 5 algorithms and the ACCV are 0.9750, 0.9360, 0.9070, 0.9180, 0.9020 and 0.9880, respectively. From the above evaluation indicators, we can see that the classification performance of ACCV also has a greater advantage compared with the comparison algorithms.

## Conclusion

In this paper, an algorithm for automatic detection and grading of cataracts using eye lens images collected by mobile phone slit lamps is proposed. The innovation of this method lies in the following: the research object of this algorithm is the horizontal scanning video of the lens. With YOLOv3 as the auxiliary positioning, it is a new method to quickly determine the position of the light knife in the pupil in YCrCb. This method solves the shortcomings of using only a single lens image in most current studies, increases the amount of input information, and is closer to the actual diagnosis process of an ophthalmologist. After the introduction of YCrCb space, the recognition speed is greatly improved, and the real-time performance of the system is promoted. Experimental results show that the calculation speed of this algorithm is much faster than purely using YOLOv3. It can precisely capture the position of the pupil in each frame, and accurately classify the light knife section of each frame in the video.

Clinically, the doctor will not judge whether the examinee is suffering from cataract based on a photo. Because the crystal is three-dimensional cystic, and a photo can only give partial information of the crystal. Therefore, the doctor will scan the front section with the slit lamp in diagnosis so as to obtain detailed crystal information. In this paper, the method uses the videos of crystal scanning with the slit lamp, and obtains more information than the traditional single picture method. And it also features more diagnostic locations, thus closer to the actual examination. Technically, YCrCb color space transformation is used on the basis of YOLO positioning to accelerate the deep learning detection. So fewer resources and less time are spent in detection, which provides a new idea for real-time detection.

Analysis shows that this method combined with a mobile phone slit lamp. The slit lamp is easy to carry and operate, can be used as a common screening tool in the community and provide accurate cataract screening services for common people.

Combined with the proposed method, community doctors can utilize the conveniency and easy-to-use of mobile slit lamp as well as extend screening to patients' homes, community health service stations, and various rural health stations. Health care workers only need to connect the mobile slit lamp, follow operation procedures to collect the crystal-scanning video, and then upload it through RUIMU-APP to the background server. With the proposed algorithm, the server can quickly send the results to the examiner's mobile phone, providing guidance to health care workers who do not have the ability to do the examination. After the primary screening, the majority of cataract patients who have not been able to go to the hospital for various reasons can be found. Then they will be referred to the professionals in hospitals for further examination, thus eliminating the missed diagnosis of other related diseases. Through the above process, the consultation rate can be improved. And Instead of going to the hospital for cataract examination, patients in areas without eye check condition or those who have not been examined due to geographical restrictions, poverty and other factors can be precisely treated.

In addition, cataract patients may also suffer from other ophthalmological diseases, which may be easier to capture in dynamic video, such as keratoconus. The degree of corneal flexion can be calculated by the dynamic changes of corneal reflection. Therefore, we will focus on extending this algorithm to the detection of other eye diseases in the future.
